# Supplementary material for: GHz acousto-optic angular momentum with tunable topological charge
Source: Nat Commun. 2025 Aug 30;16:8116. doi: 10.1038/s41467-025-63362-w (PMC12398540; doi:10.1038/s41467-025-63362-w)
Supplement: Supplementary file 1 — Supplementary Information [file 41467_2025_63362_MOESM1_ESM.pdf]

SUPPLEMENTARY INFORMATION:
Acousto-optic modulated vortex beam with tunable topological charge

A. Pitanti,[1,2] N. Ashurbekov,[1] I. de Pedro-Embid,[1] M. Msall,[1,3] and P. V. Santos[1,*]

[1] *Paul-Drude-Institut für Festkörperelektronik, Leibniz-Institut im Forschungsverbund Berlin e. V., Hausvogteiplatz 5-7, 10117 Berlin, Germany*

[2] *University of Pisa, Dipartimento di Fisica E. Fermi, largo Bruno Pontecorvo 3, Pisa 56127, Italy*[†]

[3] *Department of Physics and Astronomy, Bowdoin College, Brunswick, Maine 04011, USA*

(Dated: August 6, 2025)

Supplementary Information for the manuscript "Acousto-optic modulated vortex beam with tunable topological charge".

I. SUPPLEMENTARY NOTE 1: ACOUSTIC EXCITATION BAND

The electrical characterization of the investigated devices was performed by contacting the device via a rf probe and measuring the S_{11} scattering parameter in a wide frequency range. By evaluating the S_{11} signal in the time domain we observed the characteristics echoes originating from the multiple reflections of the acoustic wave within the high-reflectivity substrate bottom face. A spectral map of the time domain signals is shown in fig. S1 (a), which was required by recording time-domain profiles with 200 MHz bandwidth from central driving frequencies ranging from 0.2 to 18 GHz. As can be seen both from the map and from its time-integrated signal I_{S11} reported in panel (b), the main acoustic excitation band spans from about 0.5 to 7 GHz. This depends on the chosen layer thickness (700 nm of ZnO) of the bulk acoustic wave resonator (BAWR) structure.

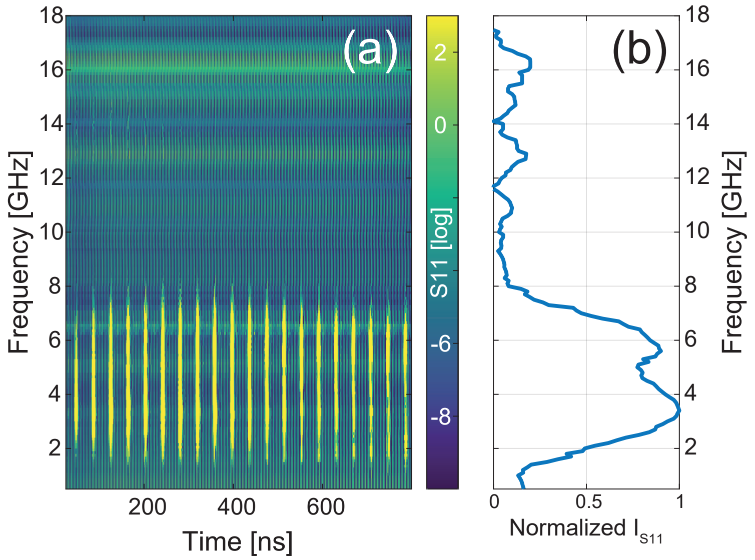

FIG. S1. (a): Wide range spectrogram of the S_{11} parameter. The main excitation band ranges from 0.5 to 7 GHz, as can be seen in the integrated intensity in panel (b).

* corresponding author: santos@pdi-berlin.de

† Also at CNR-Istituto Nanoscienze, piazza San Silvestro 12, 56127 Pisa - Italy; corresponding author: alessandro.pitanti@unipi.it

II. SUPPLEMENTARY NOTE 2: VORTEX MODELING

A. Analytical models

Interesting insights on the vortex fields can be captured by simple analytical models. Let's start by sketching the vortex as a phasor of constant amplitude and varying phase. The phasor description well catches the main vortex features: a constant ring-shaped amplitude and a set number of phase winding within a single oscillation cycle, which corresponds to a full circular revolution around the vortex physical center (parametrized by the angle θ), corresponding to the vortex topological charge. Figure S2 (a) sketches the phasor representation by considering a point rotating along a fixed orbit in the complex plane. Considering, for example, $\ell = 3$, the total phase accumulation upon one cycle (i.e., the full geometrical loop around the center) is $3 \times 2\pi$, as shown in the figure, where the phase has been wrapped in the range $[-\pi, \pi]$. Now, let's assume a simple, static background signal displacing the vortex as reported in panel (b). As one would expect, the same revolution around the vortex center would lead to 3 phase oscillations, albeit at a reduced range, not reaching the total accumulated phase found in the previous case.

A more rigorous approach considers a vortex in the polar complex plane along with a general background field both oscillating at the same (unitary) frequency:

$$w(\theta, \mathrm{Bkg}) = (e^{i\ell\theta} + \mathrm{Bkg})e^{it} \tag{1}$$

where the vortex amplitude has been normalized to 1 and Bkg is the background amplitude. Figure S2 (c) and (d) show the amplitude and phase resulting from Eq. (1) by assuming $\ell = 1$ and considering Bkg in a range from 0 (yellow curve) to 2 (dark red curve). In absence of a background signal we recover the familiar features of a regular vortex, namely a constant, ring-shaped amplitude and a 2π accumulated phase. Interestingly, an increase in the background signal introduces oscillations in the amplitude and a reduction of the phase excursion range; avoiding wrapping, the latter effects also results in the formation of oscillations for the phase, as previously discussed in the phasor sketch. Figure S2 (e,f) and Fig. S2 (g,h) reports similar calculation for $\ell = 2$ and $\ell = 3$, respectively, confirming that the presence of a background translates in modulated signals with a number of oscillations equal to ℓ within the angular range corresponding to a full revolution around the center. We decided to consider background levels from 0 to 2; note that further increasing the background would follow the same trend depicted here, reducing the total phase excursion even more.

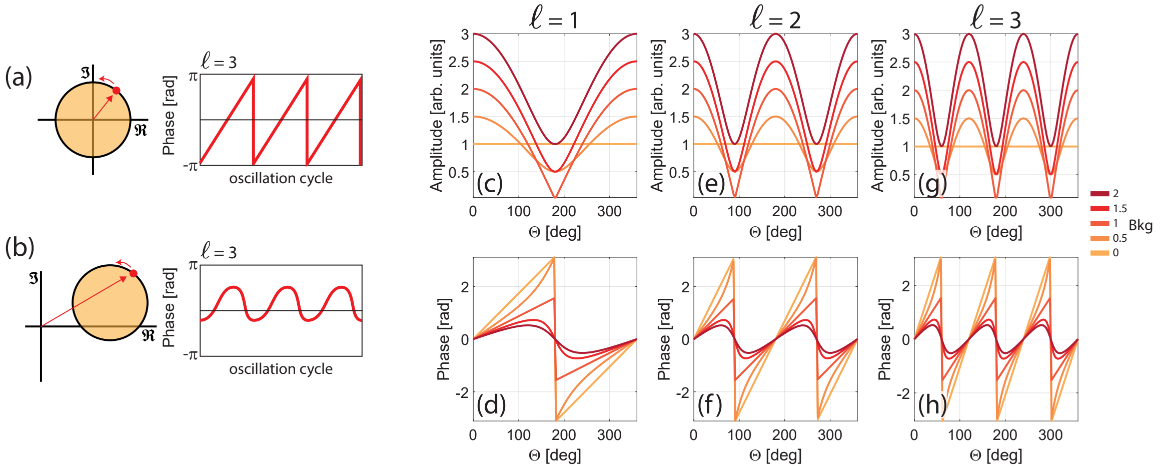

FIG. S2. (a): Sketch depicting a $\ell = 3$ vortex in the complex plane including its phase shift upon one rotation around its center. (b): Sketch of the same vortex with a constant offset. Analytical amplitudes (c,e,g) and phases (d,f,h) of vortices with topological charge 1, 2 and 3, respectively and varying harmonic offset (Bkg).

B. Numerical model

A more detailed numerical modeling of the vortex can be obtained by considering it as an additional component over the acoustic waves generated by a circular BAWR. In a first approximation, a circular BAWR can be approximated as as a drum resonator with eigenmodes given by the superposition of Bessel radial functions; as a straightforward generalization we can then imagine the overall device top surface acoustic field, described in polar coordinates in the plane $\hat{r} - \hat{\theta}$, as being composed by a background irrotational component (given by the acoustic field from a circular BAWR) plus a vortex component, obtaining the following expression:

$$w(r, \theta; B) = J(\ell, r\lambda)e^{i\ell\theta} + B \cdot J(0, r\lambda). \tag{2}$$

Here, J are Bessel functions of the first kind and λ_a the acoustic wavelength, while B is the signal amplitude of the background normalized to the vortex one. Equation (2) can be evaluated in the spatial plane to reproduce the field maps. In the first row of Fig. S3 we have plotted amplitude and phase for vortices with topological charge from 1 to 3, considering no background signal ($B = 0$). As expected, we found the familiar ring-shaped amplitude and linearly increasing phase in a loop around the center with a phase singularity at $r = 0$. Adding a background signal dramatically changes both the amplitude and the phase of the vortex, as can be seen in the other rows of Fig. S3, qualitatively agreeing with the analytical modeling and reproducing features similar to the ones observed in the experiment. In particular, the increasing background level shifts S3 (a) and shifts and splits S3 (b-c) the vortex singularity. When B becomes significantly larger than one, the field central region is completely dominated by the background, with a constant phase and an amplitude antinode; here the rotational nature of the vortex is masked by the background, although it can still be recognized in the field outer rim.

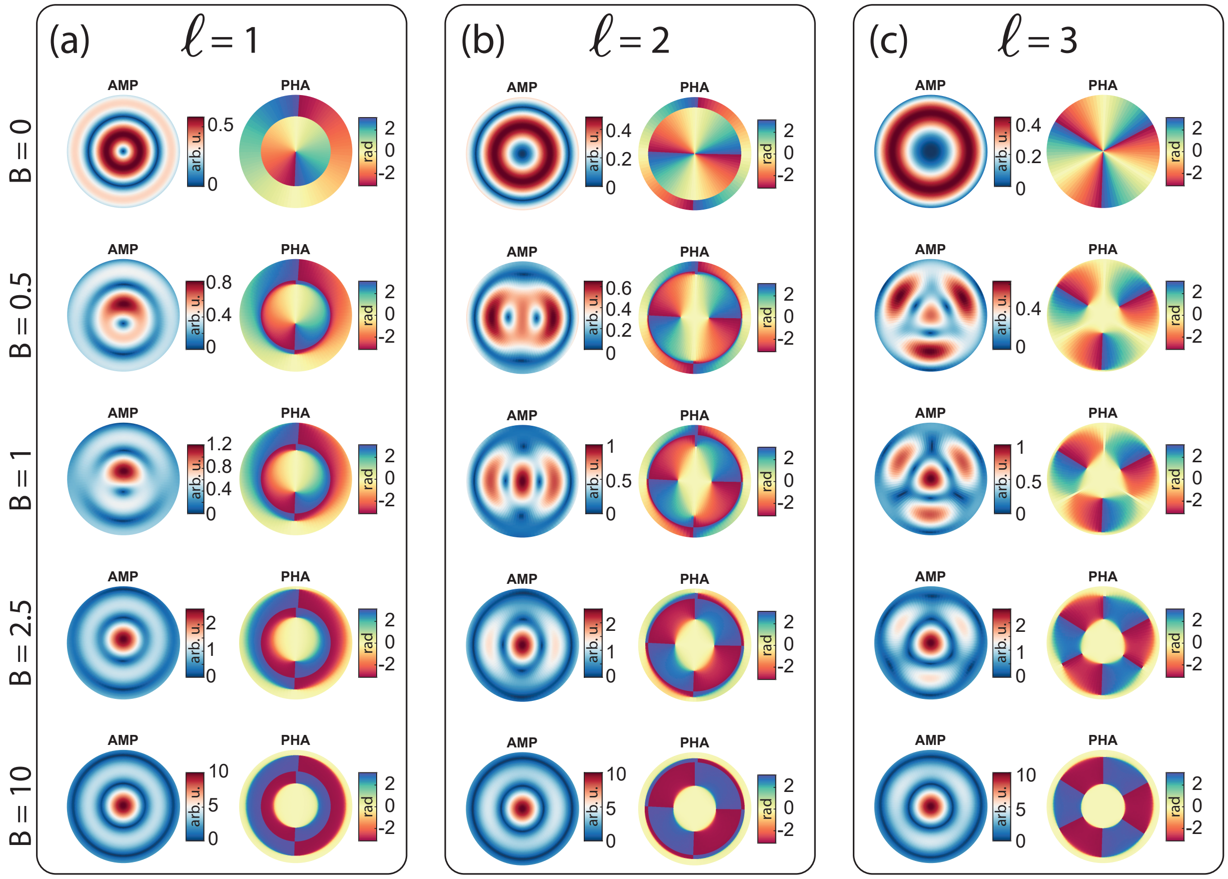

FIG. S3. Numerical polar map of field amplitude and phase in the vortex plane at different background level B. We considered vortices with different topological charges, namely $\ell = 1$ (a), $\ell = 2$ (b) and $\ell = 3$ (c), respectively

C. Acousto-optic modes orthogonality

In the main text, we discussed the main acousto-optic coupling mechanism, which relies on the phase shift of reflected light due to the out-of-plane mechanical displacement (moving boundary effect). The possibility of interferometric probing the mechanical vibrations originates from the very same mechanism, which is the most relevant one for metallic surface and vertically polarized acoustic waves as in our device. Another possibility could be given by the strain-induced refractive index modification (photo-elastic effect), which would impact more considering semiconductor surfaces. We report here for convenience the expression for the reflected field in the $\hat{r} - \hat{\theta}$ plane:

$$E_r(r,\theta) = \mathcal{R} \cdot E_i(r,\theta) e^{2\pi i \frac{2\Re[\Delta z(r,\theta)]}{\lambda_o}} \tag{3}$$

where λ_o the optical wavelength, $\mathcal{R}$ the reflectivity and E_r and E_i are reflected and incident electric field amplitude, respectively. Considering an unitary reflectivity and an illuminating planar wavefront (or at least a Gaussian beam with beam waist larger than the typical vortex size - around 5 - 20 μm in our device), we can evaluate the optical field as reflected by vortices with different ℓs. Figure S4 reports amplitude and phase resulting from Eq. (2) (mechanical field) and Eq. (3) (optical field) evaluated for $\ell = 1$, Fig. S4 (a) (also in the main text), $\ell = 2$, Fig.S4 (b) and $\ell = 3$, Fig.S4 (c), respectively. Here, we considered $\max[\Re(\Delta z(r_\theta))] = \lambda_o/4$ and no acoustic background, $B = 0$. As can be seen, the acoustic wave reproduces the familiar vortex structure, as seen in the sec. II B; the reflected field keeps a constant amplitude profile in plane and a phase modified according to its interaction with the acoustic vortex. While the reflected field is not a proper vortex beam, which can possibly be constructed within a cascaded interference scheme, it carries orbital angular momentum and rotates at the mechanical driving frequency - i.e. in the GHz range. To further analyze the optical fields, we have performed a Fourier analysis in spherical harmonics [1], where the Fourier coefficient are defined as:

$$A_l = \frac{1}{2\pi} \int_0^{2\pi} \psi(r,\theta) e^{-il\theta} d\theta \tag{4}$$

with ψ the optical complex field. As one can see in Fig. S4 (d), where Eq. (4) has been evaluated considering $\ell = 1, .., 5$ and the appropriate radial coordinate compatible with the phase structure features, optical modes generated by acoustic vortices with distinct topological charges carry different spherical harmonics, with the dominant one

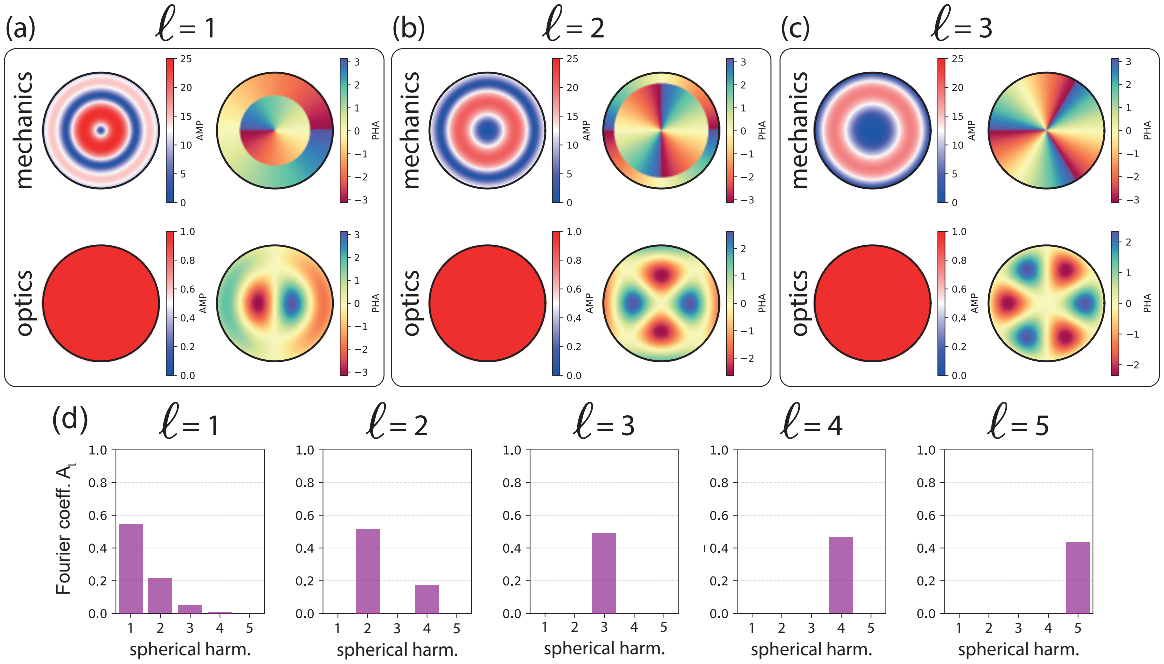

FIG. S4. Numerical polar map of the field amplitude and phase in the vortex plane considering mechanical vortices and the corresponding electric fields generated by considering perfect reflection of a planar wavefront. The maps are shown for topological charge $\ell = 1$ (a), $\ell = 2$ (b) and $\ell = 3$ (c). (d) Spherical harmonics Fourier coefficients A_l [cf. Eq. (4)] evaluated for an optical beam with plane wavefront reflected on acoustic vortices with topological charges $\ell = 1, .., 5$. The optical beam consists of a superposition of spherical harmonics with the dominant one corresponding to ℓ.

corresponding to the vortex ℓ. This proves that we can generate optical beams with a predominant OAM, which can reach high purity for in certain conditions, such as the ones in Fig. S4.

One of the most relevant characteristics of the OAM carrying or vortex beams is that they form an orthonormal base in their angular momentum/topological charge. As shown in the main text, both acoustic vortices and OAM-carrying optical beams generated in reflection configuration form orthogonal bases when different ℓs are considered. One could ask how robust is the orthogonality condition with respect to the device parameter space. Pure acoustic vortices are orthogonal by definition while the main figure of merit for the optical OAM-carrying beams is the ratio d_m/λ_o. If the acoustic displacement amplitude is one quarter of the optical wavelength the Gram matrix G_{ij}, defined as the dot product between fields with ℓ_i and ℓ_j, is practically the identity matrix (cf. Fig. 3 in the main text). Decreasing the displacement, we expect the off-diagonal terms of G_{ij} to become larger; for a small range of ℓs, we can approximate all the off-diagonal terms with the same value ε[2], making the Gram matrix a special class of a symmetric circulant matrix [3]. To quantify how much the Gram matrix differs from the identity we can then evaluate its determinant, which we can numerically calculate. Figure S5 reports the Gram matrix determinant evaluation both for mechanical and optical fields at varying acoustic maximum displacement d_m [4]. As can be seen, the determinant (det) of the mechanical Gram matrix does not depend on its own displacement and gives a constant value of 1. Conversely, the determinant of the optical Gram matrix decreases with decreasing displacement, well following the analytical expression for symmetric Gram matrices of order n:

$$\det(G) = [1 + (n-1)\varepsilon](1-\varepsilon)^{(n-1)}. \tag{5}$$

Additionally, we explicitly plotted the mechanical Gram matrices for $d_m/\lambda_o = 0.15$ and 0.3, respectively showing that, although a quasi-orthogonality of the based is conserved up to displacements of magnitude which is only roughly around one tenth of the optical wavelength. Depending on the wavelength considered, the displacement necessary could be technologically demanding: one could consider employing acoustic cavities by heterostructuring the substrate (see, for example [5]) or dedicate the use of these devices towards smaller wavelength, levaraging on the need of chiral beam manipulation and generation in the UV spectral range [6].

While increasing the overall acoustic displacement, adding substrate cavities could lead to an increase of the pervasively present acoustic background; as could be guessed from the results presented in sec. II B, a large background could also lead to a decreasing orthogonality of the acoustic vortex. Figure S5 (b) shows the latter situation, considering the mechanical Gram matrix determinant in a background range from $B = 0$ to $B = 20$, see Eq. (2). Interestingly, the addition of an irrotational background does not impact strongly on the orthogonality of the acoustic modes. Even the largest background considered, twenty-fold the amplitude of the vortex wave ($B = 20$) returns a moderate reduction of the determinant to a value slightly above 0.8, suggeseting a strong orthogonality of the modes, as can be seen by the Gram matrix in the inset.

Furthermore, the presence of the acoustic background only weakly impacts the orthogonality of the generated OAM-carrying light beams. This can be easily inferred by considering that in Eq. 3, if an incident plane wave is considered, all the angular dependence of the reflected field come from the real part of the vertical mechanical displacement $\Re[\Delta z(r,\theta)]$. If we assume a constant oscillating background, we can simply factor them out as a multiplicative constant with no angular dependence in the expression for the reflected electric field $E_r(r,\theta)$, i.e.,

$$E_r(r,\theta) = E_i e^{2\pi i \frac{2\Re[Z_0]}{\lambda_o}} e^{2\pi i \frac{2\Re[\Delta z(r,\theta)]}{\lambda_o}} = \tilde{E}_i e^{2\pi i \frac{2\Re[\Delta z(r,\theta)]}{\lambda_o}} \tag{6}$$

The background thus simply rescales the modes with an effective reflected field amplitude $\tilde{E}_i$ without affecting their orthogonality. Similar conclusions can be reached considering an azimuthally symmetric background, such as the one generated by $\ell = 0$ drum modes; in this case, we expect that the effective field amplitude will carry a radial dependence, $\widetilde{E_i(r)}$ but the angular dependence contribution will still be dependent by the acoustic vortex under consideration.

In conclusion, the orthogonality of both mechanical and optical modes obtained at different ℓs is present in a wide region of the parameter space, enabling the use of both acoustic and optical modes for advanced applications, such as light-based communication or information encoding in the mechanical waves. As a final note, some interesting device applications, such as particle manipulation and ultrahigh acceleration jet streams, do not require any orthogonality between the modes.

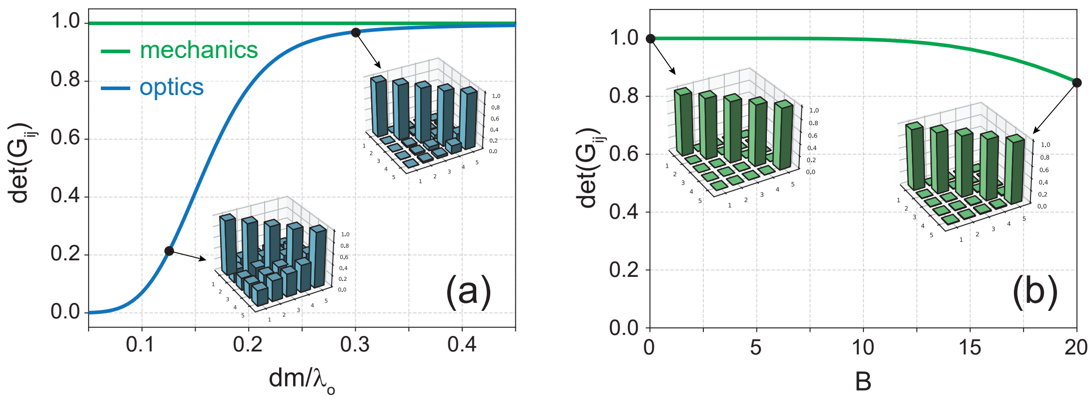

FIG. S5. (a): determinant of the mechanical and optical Gram matrices as a function of the average maximum displacement of the acoustic vortices. (b): determinant of the mechanical Gram matrix as a function of the acoustic background displacement B.

III. SUPPLEMENTARY NOTE 3: 3D FEM SIMULATIONS

The 3D simulations discussed in the main text included fully coupled electrostatic plus mechanics partial differential equation systems, which have been evaluated in composite geometrical domains using the finite element method implemented by a commercial solver (Comsol Multiphysics).
Some details on the geometrical parameters of the simulation can be found in the Methods section in the main manuscript. The material parameters are reported in the following. Sapphire, aluminum and air have been treated as isotropic materials; while sapphire domain has been included in both mechanical and electrostatic equations, the air layer has been included only in the electrostatic simulation, whereas the aluminum domains only in the mechanical one. For the latter case, being aluminum the constituent of the device electrical contacts, the voltage signal have been applied to the aluminum boundaries without including the internal material domain itself in the electrostatic simulations. The materials numerical parameters have been reported in Table S1. Finally, zinc oxide material

Material	Young's modulus [GPa]	Poisson's ratio	Density [Kg/m^3]	Relative permittivity
sapphire	400	0.3	3980	9.4
aluminum	292.6	0.335	3422.4	-
air	-	-	-	1

TABLE S1. Mechanical and electrical properties of a set of materials employed in the simulations.

has been included in both electrostatic and mechanical equation and described employing the elasticity matrix c_{ij}, the piezoelectric coupling constant e_{ij} and a relative permittivity of 7.38. The material properties have then been evaluated in a rotated reference frame with Euler's angles of $[\pi/4, 0, 0]$ to reproduce our experimenlta condition. The full expression of the unrotated matrices (in Voigt notation) are the following:

$$c_{ij} = \begin{pmatrix} 209 & 120.35 & 104.6 & 0 & 0 & 0 \\ 120.35 & 209.6 & 104.6 & 0 & 0 & 0 \\ 104.6 & 104.6 & 210.6 & 0 & 0 & 0 \\ 0 & 0 & 0 & 42.3 & 0 & 0 \\ 0 & 0 & 0 & 0 & 42.3 & 0 \\ 0 & 0 & 0 & 0 & 0 & 42.3 \end{pmatrix} [GPa],\ e_{ij} = \begin{pmatrix} 0 & 0 & 0 & 0 & -0.48 & 0 \\ 0 & 0 & 0 & -0.48 & 0 & 0 \\ -0.573 & -0.573 & 1.321 & 0 & 0 & 0 \end{pmatrix} [C/m^2] \tag{7}$$

As described in the Methods section in the main manuscript, nominal geometrical parameters have been employed for all the constituents except the sapphire substrate whole length was reduced to 25 μm.

IV. SUPPLEMENTARY NOTE 4: GEOMETRICAL TUNING OF TOPOLOGICAL CHARGE

We have employed our 3D FEM simulation platform to demonstrate the tuning of ℓ with the device geometry, which we explored considering a range for the parameter g from $g = 0.1\ \mu m$ to $g = 16\ \mu m$ in the $M = 1$ spiral, refs. to Eq. (1) in the main text.

The main results of the frequency-domain simulations are reported in Fig. S6. The simulated frequency choice is of 1.134 GHz, which is characterized by a moderate background signal resulting in an improved vortex visibility. The spiral contact for the smallest g can be easily approximated as a circular BAWR, expecting solutions proportional to circular Bessel functions. This is indeed well-reproduced, as can be seen by inspecting the real part of the out-of-plane displacement field component w, evaluated at the device top surface and reported as the map in the first row of Fig. S6(a) (first column). As a well-known fact, ordinary radial Bessel functions do not generate any rotational component, in fact the same result can be obtained with the previously discussed analytical model, considering Eq. (2) for $\ell = 0$, (second column). The irrotational nature of the field can also be appreciated by evaluating the vectorial

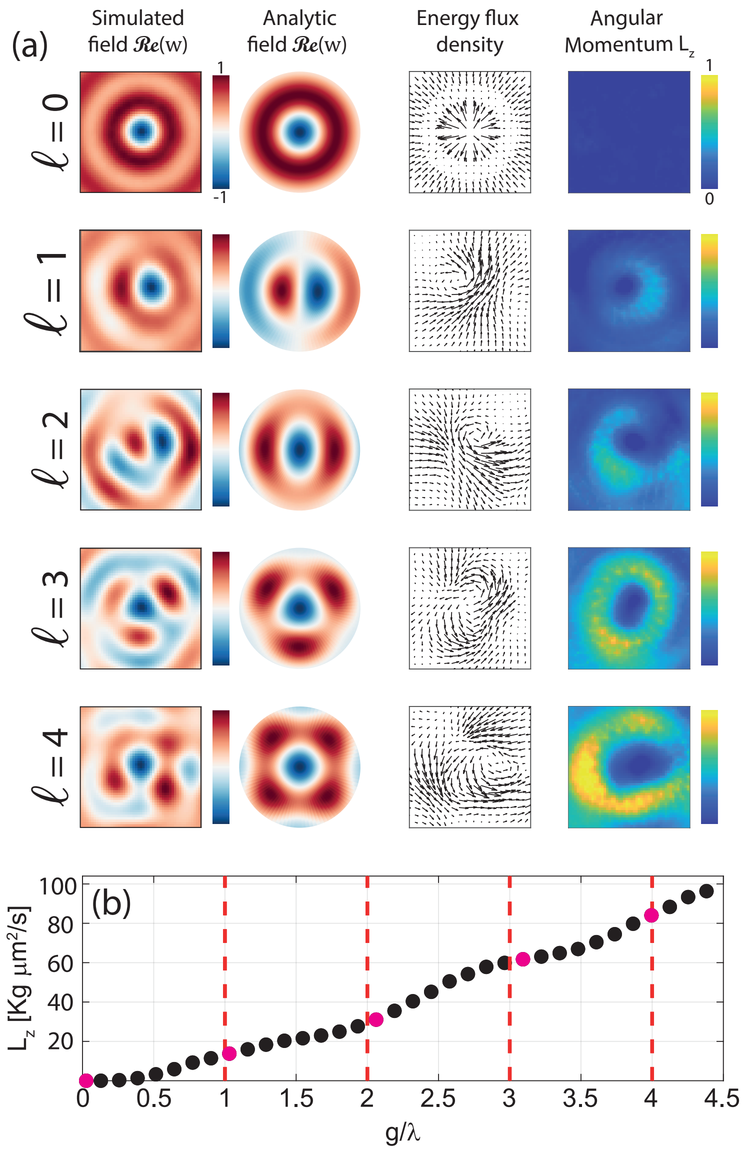

FIG. S6. (a): Selected maps of simulated acoustic field components (first column), energy flux density (third column) and total out-of-plane angular momentum (L_z, cf. Eq. 8). (b) Dependence of L_z on the spiral spoke length g for single spoke spiral. The red-colored dots indicate the points where the maps in panel (a) have been extracted. The solid line is a linear fit to the data, showing a smooth increase of L_z with g.

.

energy flow in the device plane $\vec{\Phi}$, which shows no rotational component (third column), leading to a vanishing total out-of-plane angular momentum (L_z, fourth column). L_z was evaluated via the integral

$$L_z = \int_V \vec{r} \times \vec{\Phi}\, dV, \tag{8}$$

where $l_z = \vec{r} \times \vec{\Phi}$ is the volumetric angular momentum density and V is the three-dimensional volume of the device. This expression can be derived considering the energy flux density as the acoustic counterpart of the Poynting vector for electromagnetic waves, see [7, 8]. In our specific case, the angular momentum density has been evaluated within the sapphire substrate and then integrated over the three-dimensional space to obtain the total angular momentum L_z. Finally, the $\ell = 0$ results can be used to extract the acoustic wavelength, fitting the spatial map profile with Eq. (1). The obtained wavelength of $\lambda = 3.5$ μm, which represents the system length scale and can suggest the resonant condition for a vortex formation, i.e. $\eta = g/\lambda = \ell$, cf. discussion in connection with Fig. 4 of the main article.

As expected, increasing the value of g leads in fact to profound changes in the acoustic fields. We can clearly recognize the appearance of vortices with different topological charges, from 1 to 4 in the simulated range (see Fig. S6(a)). The numerically evaluated fields are in good agreement with the analytical ones; moreover, the energy flux density shows a clear rotation around the center, leading to a net angular momentum along the vortex ring.

The overall L_z trend can be inspected in Fig. S6 (b), where the colored dots indicate the points where we extracted the maps reported in panel (a). The overall trend of L_z is linear and smoothly increasing with g; the continuity of the function and its first derivative give no indication of quantization, suggesting instead the overlapping of broadband vortex resonances centered at the integer values of the η value.

V. SUPPLEMENTARY NOTE 5: ADDITIONAL INFORMATION ON OPERATING FREQUENCY BAND

Considering our experimental bench and device characteristics, the frequency range we where we can characterized the vortex fields goes from about 400 MHz to 2 GHz, limited by the interferometer detector cut-off frequency ($\sim$ 1 GHz) and light diffraction limit. Figure S7 (a) shows the full amplitude spectrum of the vibration acquired at the device center, where we purposely defocused the laser beam to obtain a wide probed area. The sharp peaks with a free-spectral-range of about 13 MHz correspond to the longitudinal wave resonances within the substrate, as discussed in the main text. They arise from an oscillating background that can be ascribed to both degeneracy breaking the BAWR drum modes as well as having a non-calibrated measurement. As a supplementary measurement of a vortex at high frequency, Fig. S7 (b) reports the phase profile obtained at 1326 MHz and showing a $\ell = 4$, complementing the results reported in Fig. 3 in the main manuscript.

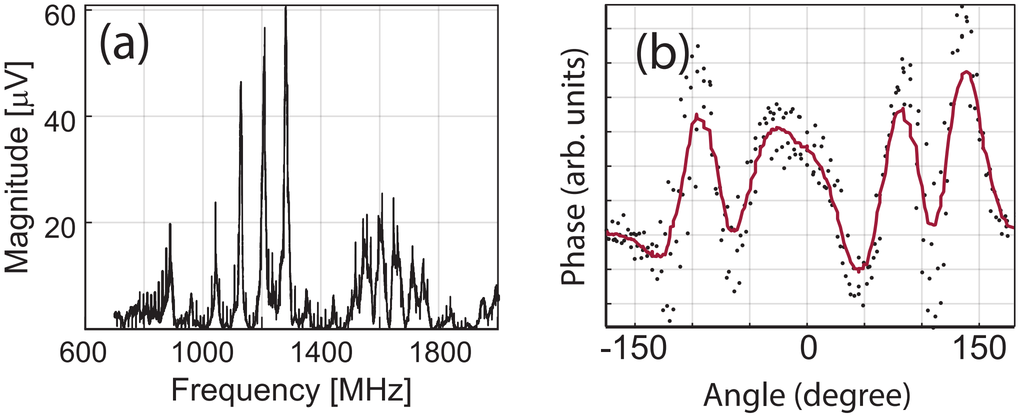

FIG. S7. (a): Experimental vibration amplitude in the device center in a wide frequency range. (b): Phase profile of a vortex with $\ell = 4$

VI. SUPPLEMENTARY NOTE 6: SIMULATION OF DEVICES WITH INCREASING NUMBER OF SPOKES

In the main text, we have shown how simple design modification can be used to obtain vortices with higher topological charges at the same driving frequency. As an example, Fig. 5 in the main text shows the phase evolution on a circular path around the vortex center for devices having $M = 1, 2$ and 3, respectively, cf. Eq. (4) in the main text. While driven at the same frequency of 1158 MHz, the three devices show a topological charge which linearly scales with the spoke number M, as can be guessed by the increasing number of peaks in the measured phase profile. Figure S8 shows the respective simulations for the three investigated devices: as in the experiment, we can see 3 peaks for the $M = 1$ device (a), 6 peaks for the $M = 2$ (b) and finally 9 peaks for the $M = 3$ one (c), strengthening the experimental claims reported in the main text. Even in this case, the different range of phase excursion have to be imputed to the different substrate thickness used in the simulation model.

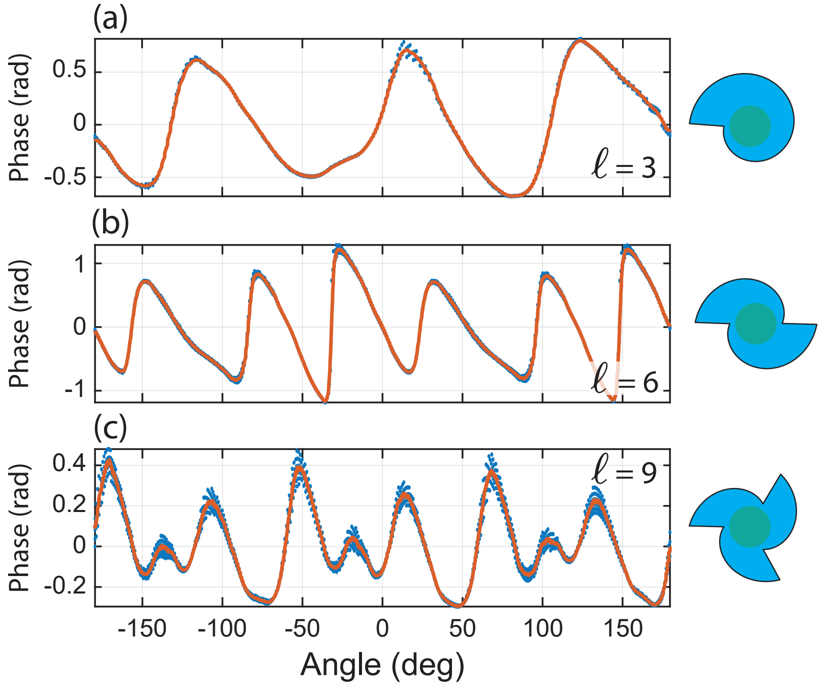

FIG. S8. Simulated phase profiles around the vortex center for devices with $M = 1$ (a), $M = 2$ (b) and $M = 3$ (c) for the same driving condition of Fig. 3 in the main text.

VII. SUPPLEMENTARY NOTE 7: SUBSTRATE EFFECTS ON ACOUSTIC WAVE PROPAGATION

Some interesting insights can be obtained investigating the propagation of the acoustic beam within the substrate. This can only be done numerically, since our experimental technique is sensitive only to the acoustic fields close to the device top surface. Let's start by considering that the BAWR launches a set of acoustic waves with varying wavevectors, which combine to form Lamb modes, as discussed in the main text. One can imagine that Lamb modes form upon multiple reflection of an angled longitudinal wave component within the substrate, suggesting that a polished bottom surface is important to excite the laterally propagating modes. The wave propagating directly below the BAWR is thus expected to be subjected to a certain degree of divergence, increasing the size of the features (such as the vortex) propagating towards the substrate bottom face. Moreover, a possible flipping of the wave helicity upon reflection should be considered, as routinely seen for example in electromagnetic waves reflected from a mirror.

To investigate possible detrimental contributions due to these effects, we checked the acoustic field in $\hat{x} - \hat{y}$ planes at different substrate depths. Figure S6 shows a typical simulation for a vortex with $\ell = 3$ where the 25 μm thick substrate has been stretched to improve the visibility of the different slices. As can be seen, the vortex beam is present over the whole substrate length, although its signature features (i.e. 3 lobes) are less visible in proximity of the substrate bottom face, due to the effects previously described. Overall a very clear vortex structure can be recognized well below the substrate half depth; in particular we reported the planar energy flux in the figure right

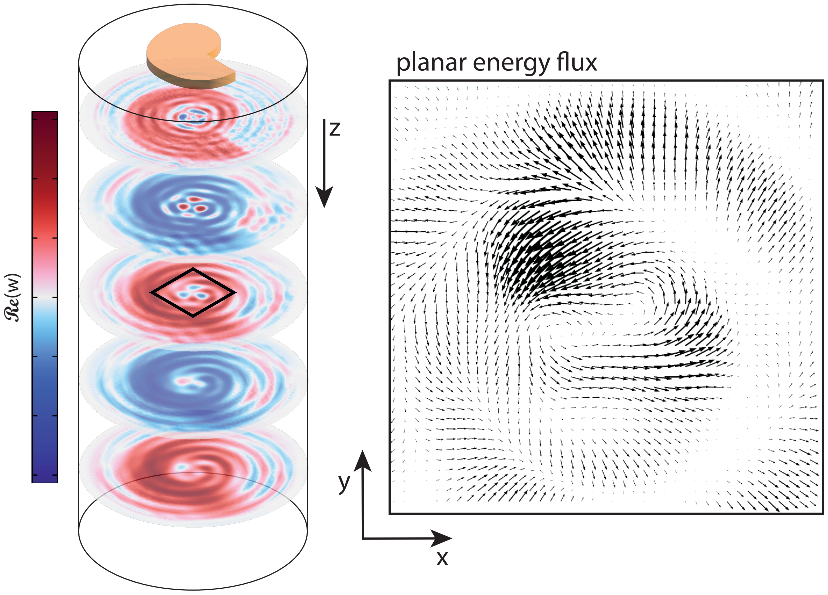

FIG. S9. Simulated real part of the acoustic field at different depths (not in scale). The details of the energy flux at the substrate half depth is reported in the inset.

panel, showing a net rotational energy flow.

As an additional simulation, we reiterate the role of the substrate reflectivity in reducing the phase range of the acoustic vortex. Figure S10 reports the simulated vertical displacement phase map at the device top surface at a driving frequency of 1.121 GHz. As reported in the main text, a standard simulation comprising a fixed boundary at the substrate bottom face shows a limited phase excursion, about 1.8 rad for the simulation depicted in Fig. S10 (a). The hypothesis that this is due to the presence of background signal enhanced by the perfect reflectivity of the wave at bottom surface can be easily verified by changing the boundary conditions. In facts, when the substrate bottom face is simulated using a perfectly matched layer to absorb the incoming wave, the full, 2π rad phase range is restored, as shown in Fig. S10 (b), demonstrating the strong impact of the substrate on the presence of the acoustic background. A further improvement can be obtained by considering a different patterning of the Archimedean spiral top contact.

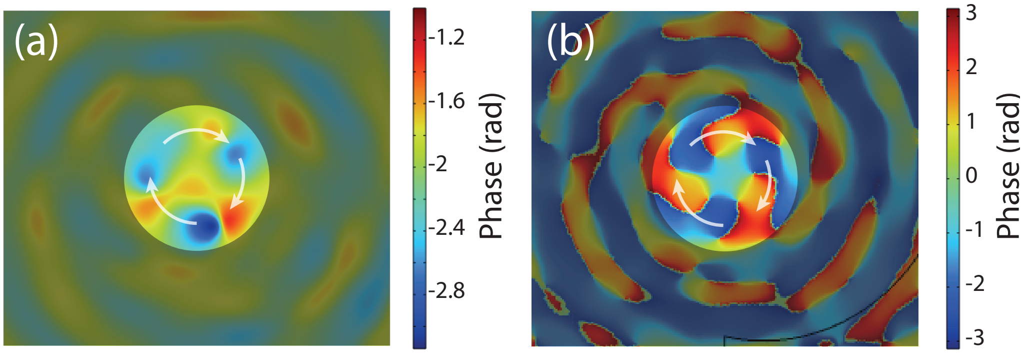

FIG. S10. Simulated phase map at the device top surface at a driving frequency of 1.121 GHz for a reflecting (a) and absorbing (b) bottom face.

Switching from a filled area to spiral-shaped finger contacts suppress the generation of $\ell = 0$ modes as well as forcing a resonant condition of the waves propagating in the $\hat{r} - \hat{\theta}$ plane. Figure S11 reports FEM simulations of such a structure, considering the same simulation cell of the main text, a non-reflecting substrate back face and a differently shaped BAWR top contact. At a driving frequency of 500 MHz, we obtain the generation of a clear $\ell = 1$ vortex with

the well known ring-shaped amplitude (Fig. S11 a), the central singularity and the monotonically increasing phase from from $-\pi$ to π radians in a loop around the center (Fig. S12 b), further strengthening the results of Fig. S10.

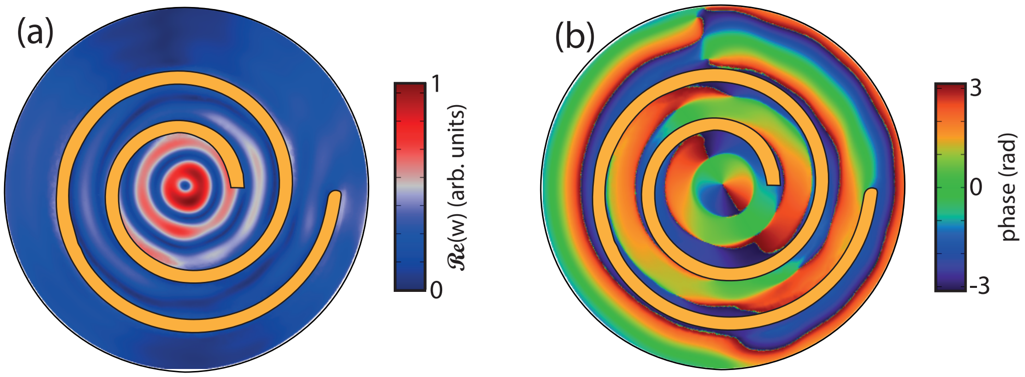

FIG. S11. FEM simulation of an acoustic vortex generated via spiral-shaped fingers top contact. (a) - amplitude and (b) phase maps. simulation cell size is the same as in the main text, the substrate back face has been considered perfectly absorbing introducing a PML layer.

VIII. SUPPLEMENTARY NOTE 8: FORK INTERFEROMETRY

Fork interferometry is a well-established technique to measure the phase profile of an optical wavefront with OAM. As for the scanning interferometric measurements described in the main text (cf. Figure 2 of Ref. 9), fork interferometry can be implemented using a Michelson setup to interfere the impinging optical beam with OAM with a reference beam with linearly varying phase profile. Contrary to scanning interferometry, the impinging optical beam needs to be expanded to cover the whole area of acoustic vortex. The linearly varying phase gradient can be imposed on the reference beam by slightly tilting the reference mirror to create interference fringes. Alternative configurations can also be implemented, e.g., by first splitting the AOM-carrying beam, introducing a phase shift, and then properly interfering the splitted beams. In all cases, the resulting interference pattern exhibits a lateral shift of the interference lines in the acoustic vortex area, which is proportinal to the OAM transferred to the reflected optical beam. For an $\ell = 1$ beam, the shifts appears as a fork-like pattern indicative of the presence of a vortex.

The previous considerations apply for a beam with time-constant AOM. For the time-dependent phase shifts induced by an acoustic vortex, the time-integrated interferometric pattern becomes smeared out. Sharp pattern for AOM determination need, therefore, to be acquired with time resolution much shorter than the acoustic period. Alternatively, one can employ a stroboscopic measurement technique using a pulsed laser beam with a repetition rate locked to an integer submultiple of the acoustic frequency.

The fork interferogram pattern $I_{\mathrm{fork}}(x, y, t)$ can be easily determined by assuming that the acoustic vortex with a Gaussian waist σ induces a phase modulation $\Delta_{\mathrm{ph}}(x, y, t)$ of phase span $\Delta_{\mathrm{ph},0}$. For a $\ell = 1$, $\Delta_{\mathrm{ph}}(x, y, t)$ is given by:

$$\begin{aligned} \Delta_{ph}(x, y, t) &= \Delta_{ph,0}\theta e^{-\left(\frac{r}{\sqrt{2}\sigma}\right)}, \quad \text{where} \\ r &= \sqrt{x^2 + y^2} \\ \theta &= \arctan\frac{x}{y} + \omega_a t, \end{aligned} \tag{9}$$

and where ω_a is the angular frequency of the acoustic wave. If the diameter of the optical beams are much larger than the vortex radius σ, one obtains:

$$I_{\mathrm{fork}}(x, y, t) = \frac{1}{2}\left[e^{\imath(k_{grad}x - \omega_o t)} + e^{\imath(\Delta_{\mathrm{ph}}(x,y,t) - \omega_o t)}\right] \tag{10}$$

where ω_o is the optical angular frequency. The first term within the square brackets is the reference beam with a phase gradient $k_{grad}x$ assumed to be along the x direction of the beam cross-section. The second term represents the beam reflected on the vortex, which acquires a cross-sectional phase profile given by Eq. 9.

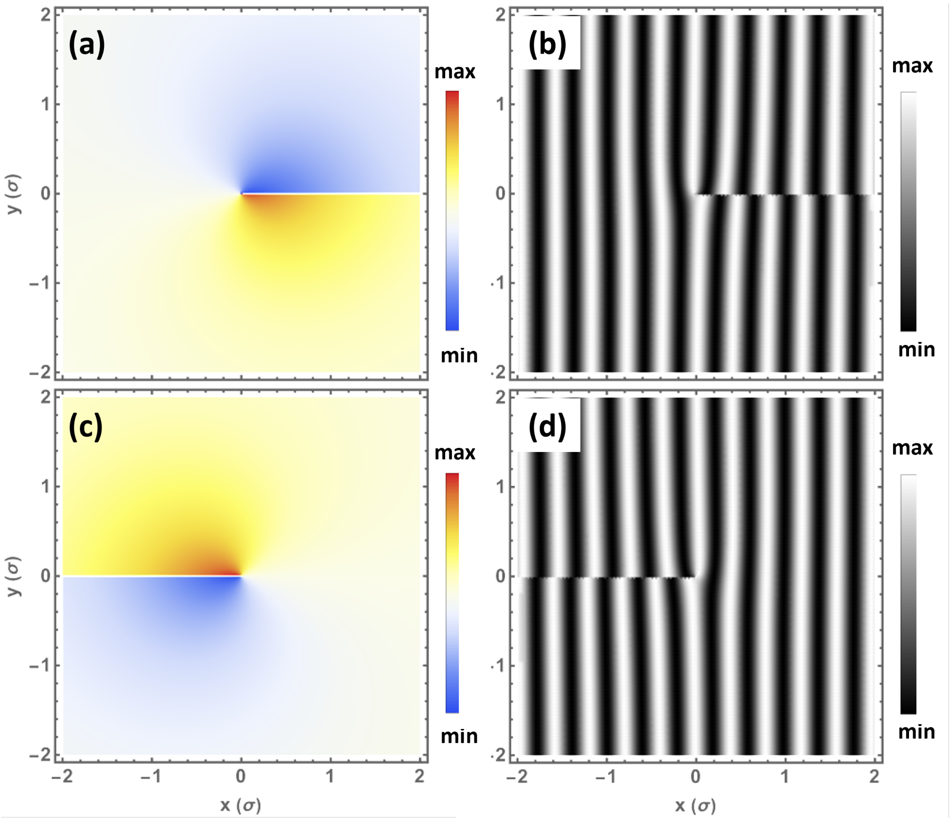

FIG. S12. (a) Phase modulation $\Delta_{\rm ph}(x,y,t)$ (cf. Eq. 9) induced by an acoustic vortex with $\ell = 1$ and (b) corresponding fork interferogram $I_{\rm fork}(x,y,t)$ (cf. Eq. 10) calculated for $t = 0$. The fork pattern is characterized by a lateral shift of the interference fringes at the vortex site, which is best seen at the phase discontinuity in $\Delta_{\rm ph}(x,y,t)$. The calculations were performed assuming a phase span $\Delta_{\rm ph,0} = 3$ rad equal to the largest measured in the experiments (cf. Fig. 4(a) of the main text) and $k_{grad}\sigma = 16$. (c) and (d) are the corresponding plots for a time instant delayed by half an acoustic periond (i.e., for $t = \pi/\omega_a$).

Figures S12(a) and S12(b) display the imprinted phase $\Delta_{\rm ph}(x,y,t)$ and the corresponding fork interferogram calculated for fixed time instant $t = 0$. The phase span $\Delta_{\rm ph,0} = 3$ rad, defined as the phase excursion around the vortex, was taken to be equal to the largest one measured in our sample (cf. Fig. 4(a) of the main text). The fork pattern is characterized by a lateral shift of the fringes, which can be better identified at the phase discontinuity in $\Delta_{\rm ph}(x,y,t)$. Under an acoustic modulation, the location of the shifts (and discontinuities) changes with time. As an example, Figs. S12(c) and (d) show the same patterns calculated after a delay of half an acoustic period (i.e., for $t = \pi/\omega_a$). In a time-integrated interferogram, the vortex will then appear as a blurred pattern centered at its location.

While our analytical estimates suggest that indeed fork-grating interferometry could be used to directly measure the time-dependent OAM of reflected light, the practical measurement present noteworthy challenges, even for a large (i.e., close 2π). Note that in most of the reported experiments the phase span is much larger that the one used in Fig. S12 A main difficulty arises from the dynamical nature of our system, which requires imaging at GHz rated and above or, alternatively, stroboscopic synchronization with pulsed lasers, which normally operate at much smaller repetition frequencies. Conversely, our interferometric technique, while not giving a direct AOM measurement of the full beam at once, allows us to reconstruct, point-by-point, the complete phase map carrying the same information as time-resolved fork interferometry.

[1] B. Jack, M. Padgett, and S. Franke-Arnold, Angular diffraction, New Journal of Physics **10**, 103013 (2008).

[2] The limit of the approximation mainly depends on the different maximum amplitude of the Bessel's functions of the first kind, which decreases with the function order, i.e. the acoustic vortex topological charge.

[3] R. M. Gray *et al.*, Toeplitz and circulant matrices: A review, Foundations and Trends® in Communications and Information Theory **2**, 155 (2006).

[4] Here to simplify the discussion, we report the average maximum displacement for the first 5 modes with increasing topological charge, $\ell = 1, .., 5$.

[5] A. S. Kuznetsov, K. Biermann, A. A. Reynoso, A. Fainstein, and P. V. Santos, Microcavity phonoritons–a coherent optical-to-microwave interface, Nat. Commun. **14**, 5470 (2023).

[6] L. Rego, K. M. Dorney, N. J. Brooks, Q. L. Nguyen, C.-T. Liao, J. San Román, D. E. Couch, A. Liu, E. Pisanty, M. Lewenstein, *et al.*, Generation of extreme-ultraviolet beams with time-varying orbital angular momentum, Science **364**, eaaw9486 (2019).

[7] L. Zhang and P. L. Marston, Angular momentum flux of nonparaxial acoustic vortex beams and torques on axisymmetric objects, Physical Review E **84**, 065601 (2011).

[8] K. Y. Bliokh and F. Nori, Spin and orbital angular momenta of acoustic beams, Phys. Rev. B **99**, 174310 (2019).

[9] M. M. De Lima Jr. and P. V. Santos, Modulation of photonic structures by surface acoustic waves, Rep. Prog. Phys. **68**, 7 (2005).
