# Supplementary material for: GHz acousto-optic angular momentum with tunable topological charge
Source: Nat Commun. 2025 Aug 30;16:8116. doi: 10.1038/s41467-025-63362-w (PMC12398540; doi:10.1038/s41467-025-63362-w)
Supplement: Supplementary file 2 — Description of Additional Supplementary Files [file 41467_2025_63362_MOESM2_ESM.pdf]

Description of Additional Supplementary Files

Supplementary Video:

Measured (left) and simulated (right) temporal evolution of the surface displacement corresponding to the conditions in Figs. 1d and 1f.
