# Supplementary figures and images for: GHz acousto-optic angular momentum with tunable topological charge
Source: Nat Commun. 2025 Aug 30;16:8116. doi: 10.1038/s41467-025-63362-w (PMC12398540; doi:10.1038/s41467-025-63362-w)

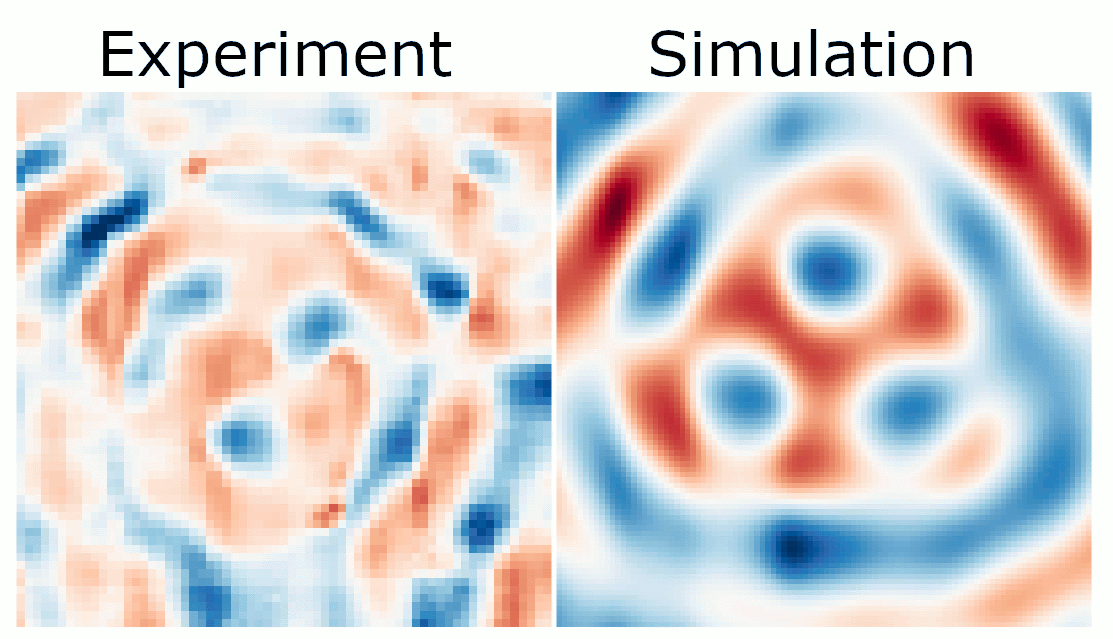

Supplement: Supplementary file 3 — Supplementary Video [file 41467_2025_63362_MOESM3_ESM.gif]
